# Targeted Recombinant Progeny: a design for ultra-high resolution mapping of Quantitative Trait Loci in crosses between inbred or pure lines

**DOI:** 10.1186/s12863-015-0206-z

**Published:** 2015-07-07

**Authors:** Eliyahu M Heifetz, Morris Soller

**Affiliations:** JCT - , Lev Academic Center, 21 Havaad Haleumi, Jerusalem, Israel; Department of Genetics, Silverman Life Sciences Institute, Edmund Safra Campus, The Hebrew University of Jerusalem, 91904 Jerusalem, Israel

**Keywords:** QTN, QTL, High resolution mapping, Inbred lines, F_2_ mapping design, Polygenic variation

## Abstract

**Background:**

High-resolution mapping of the loci (QTN) responsible for genetic variation in quantitative traits is essential for positional cloning of candidate genes, and for effective marker assisted selection. The confidence interval (QTL) flanking the point estimate of QTN-location is proportional to the number of individuals in the mapping population carrying chromosomes recombinant in the given interval. Consequently, many designs for high resolution QTN mapping are based on increasing the proportion of recombinants in the mapping population.

The "Targeted Recombinant Progeny" (TRP) design is a new design for high resolution mapping of a target QTN in crosses between pure, or inbred lines. It is a three-generation procedure generating a large number of recombinant individuals within a QTL previously shown to contain a QTN. This is achieved by having individuals that carry chromosomes recombinant across the target QTL interval as parents of a large mapping population; most of whom will therefore carry recombinant chromosomes targeted to the given QTL. The TRP design is particularly useful for high resolution mapping of QTN that differentiate inbred or pure lines, and hence are not amenable to high resolution mapping by genome-wide association tests.

**Results:**

In the absence of residual polygenic variation, population sizes required for achieving given mapping resolution by the TRP-F_2_ design relative to a standard F_2_ design ranged from 0.289 for a QTN with standardized allele substitution effect = 0.2, mapped to an initial QTL of 0.2 Morgan to 0.041 for equivalent QTN mapped to an initial QTL of 0.02 M. In the presence of residual polygenic variation, the relative effectiveness of the TRP design ranges from 1.068 to 0.151 for the same initial QTL intervals and QTN effect. Thus even in the presence of polygenic variation, the TRP can still provide major savings. Simulation showed that mapping by TRP should be based on 30-50 markers spanning the initial interval; and on at least 50 or more G_2_ families representing this number of recombination points,.

**Conclusions:**

The TRP design can be an effective procedure for achieving high and ultra-high mapping resolution of a target QTN previously mapped to a known confidence interval (QTL).

## Background

Genetic analysis of complex quantitative traits involves mapping the polymorphic sites (QTN – Quantitative Trait Nucleotide) responsible for genetic variation in these traits to their individual chromosomal regions (QTL – Quantitative Trait Loci). The QTL is defined here as a confidence interval (CI) within which the QTN is located with confidence (1-α), where α is the probability that the QTN is found outside the CI. High resolution QTL mapping (i.e., localizing the QTL to a narrow confidence interval) is essential for positional cloning of candidate genes, and for effective marker assisted selection or marker assisted introgression. With the advent of high density SNP microarrays, high resolution mapping of segregating QTN within populations of outcrossing species (all animals, and many plants), is now based on marker-QTN association due to population-wide linkage disequilibrium (LD), so-called “Whole Genome Association Studies” (WGAS) [1]. WGAS exploits for mapping the very large number of recombination events accumulated across an outcrossing population over the untold generations of sexual reproduction since the origin of the QTN. These recombination events limit population-wide LD between markers and QTN in the population to a very narrow region about the QTN, so that statistically significant association between marker and QTN indicates that the QTN is very close to the marker. Within pure lines of selfing species, or inbred lines of outcrossing species, however, markers and QTN are at fixation. Hence, WGAS is clearly not possible and other designs have been developed. Many of these were already reviewed in the classic Darvasi paper [2]. Basically they fall into two types: Group I, designs that increase mapping resolution across the entire genome; Group II, designs that are targeted to a specific QTL. Group I designs include the Advanced Intercross Line (AIL) design [3,4] which can be applied *ad hoc* to any F_2_ population. These designs also include specialized mouse stocks based on the AIL principle, developed specifically for high resolution mapping, the Heterogeneous Stock (HS) [5] and the Diversity Outcross [6]. In a happy stroke of good fortune, the commercially available MF1 outbred stock, was adventitiously found to be highly suitable for Group I mapping [7,8]. All three stocks have the advantage that mapping is on the basis of founder haplotypes instead of single markers, in this way avoiding dilution of effects when the same marker allele is associated with both alternative QTN alleles. On the other hand they all have the limitation that they can only access QTN that are segregating among the founder lines of the crosses. Resources comparable to the HS, Diversity outcross and MF1 are not available for plant species. In the Near Isogenic Line (NIL) design, analyzed in detail in [9], the entire genome of a target donor line is dissected into smaller segments, each isolated in a different NIL through successive backcrossing to a recipient line followed by a number of selfing generations. Each segment can then be tested against the corresponding donor segment for QTL detection. Construction of NIL libraries was pioneered by the tomato geneticists [10,11] and has since been applied with success in a wide assortment of plant species (listed in [9]). Also in Group I are new major resources consisting of sets of many Recombinant Inbred Lines (RIL) derived from intercrosses among a large set of founder parent lines chosen to maximize genetic diversity within the resource. These include the Collaborative Cross (CC) Mouse Resource population [12,13]; and the conceptually identical Multi-parent Advanced Generation Inter-Cross (MAGIC) resources in plants [14]. The MAGIC resource was first proposed by Cavanough [14], based on the discussions leading to the CC resource, and has already been applied to develop MAGIC populations in Arabidopsis [15], wheat [16] and rice [17]. The advantages and strengths of the CC and MAGIC resources are too many to detail here. When a large mapping population can be generated and genotyped but the limiting factor is phenotyping (as in microarray experiments), selective phenotyping (reviewed in [18]) can be employed to select a subset of individuals to maximize their mapping power, e.g., by maximizing their genotypic dissimilarity [19] or the complementariness of crossover sites within the sample [20].

Group II designs are aimed at high resolution mapping targeted to a specific QTL previously mapped with high power to a relatively large CI by standard F_2_, BC, or AIL QTL-mapping designs. Group II designs were reviewed by Darvasi [2], and new designs do not seem to have been proposed since then. All of these designs are based on the principle of “Chromosome Dissection” pioneered by the Drosophila geneticists over half a century ago (e.g., [21]). In Recombinant Progeny Testing (RPT) [2, 22] a series of individuals in the original F_2_ or BC mapping population that carry recombinant chromosomes with the points of recombination laddered across the target region are queried individually as to whether the QTN location is upstream or downstream of the recombination point. In the Interval-Specific Congenic Strains (ISCS) design [23] the same class of individuals as for RPT are individually backcrossed repeatedly to one of the parental strains (chosen to be recessive for the QTL effect, if applicable) to retain only a segment containing the recombination point and small flanking donor intervals. This establishes a series of congenic strains that cover the target region. These are then queried individually to determine whether the QTN is located upstream or downstream of the recombination point. A great advantage of the ICSC design is the reduction in residual genetic variance by the repeated backcrossing to the inbred parent. This enables strain status with respect to the QTL to be determined with relatively few individuals. This design has been widely applied in mice (e.g., [24]). When large numbers of individuals are available for the mapping population, selective recombinant genotyping [25] can be employed to reduce genotyping costs.

In the present study, we propose a new, "Targeted Recombinant Progeny" (TRP) design for high and ultra-high resolution mapping of specific target QTN that are at fixation within populations, but differ across populations, e.g., in crosses between pure lines of selfer species, or between highly inbred lines. The TRP design is a three-generation procedure for generating a large number of recombinant individuals within a QTL shown by previous mapping to contain a QTN. Similar to RPT and ISCS this is achieved by identifying “founder” individuals in an F_2_, BC, or AIL population that carry chromosomes that are recombinant in the target QTL. In contrast to RPT, there is no attempt to determine QTN status of individual founders. Rather, by having these founder individuals serve as parents of a large progeny population, a mapping population is generated most of whose members carry recombinant chromosomes targeted to the given QTL. Since the TRP mapping population consists primarily of recombinant individuals, this provides for high resolution mapping of the QTN within its QTL, with appreciably smaller total mapping populations than required for equal precision by classical F_2_, BC or even AIL designs. The only condition is that the number of founder individuals is sufficient to provide enough points of recombination to refine the QTN location to the desired degree. It should be stressed, that the TRP design is aimed at high resolution mapping of a specific QTN previously mapped to a QTL. This contrasts to the classical F_2_, BC and AIL designs for which a single set of progeny provides the required recombinants for mapping all QTN that are segregating in the mapping population.

## Methods

### (i) Notation and assumptions

#### Notation

To simplify derivations, map distance are given in units of Morgans (M), rather than centiMorgans (cM). Thus, 20 cM = 0.2 M.

#### Assumptions

We assume a QTN that has been previously mapped to a point location within a QTL of width C M, with confidence level (1-α). The purpose of the TRP is to generate a new mapping population which is densely populated by recombinant chromosomes targeted to this QTL, so that a new point location with (1-α) CI of width c = C/s is obtained, where s is the factor by which the original CI is reduced. The TRP achieves this by generating an F_2_, BC or AIL population and identifying within it individuals that carry a recombinant chromosome in the target interval. These are then selfed or backcrossed to one of the parental lines to generate a TRP mapping population highly enriched with recombinant chromosomes in the interval of interest.

**Figure 1:**
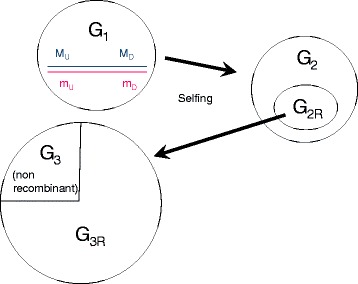
Construction of a TRP mapping population. We assume a QTN mapped to a known confidence interval bounded by markers M_U_ and M_D_. Construction of the TRP mapping population begins with one or more G_1_ individuals heterozygous for alternative alleles at the QTN and for a large series of markers spanning the interval from M_U_ to M_D_. The G_1_ individuals are selfed, generating a G_2_ population. The G_2_ population is genotyped for the markers M_U_ and M_D_ identifying a subset of individuals (the G_2R_ population) that carry a recombinant chromosome in this region together with one of the parental haplotypes . The G_2R_ individuals are genotyped for the full set of internal markers, identifying the point of recombination of their recombinant chromosome. They are selfed in turn to generate the G_3_ population. The G_3_ individuals are genotyped for one of the markers heterozygous in their G_2R_ parent to identify the haplotypes transmitted by the G_2R_ parent. G_3_ individuals carrying one or two recombinant haplotypes (75% of the total) form the G_3R_ mapping population. Non-recombinant G_3_ individuals that carry only parental type haplotypes serve to correct for polygenic family effects

It is convenient to describe the TRP by starting with an F_1_ generation created by crossing two pure lines homozygous for alternative alleles at a large number of marker loci and at the target QTL. However, the TRP can equally be initiated from any population (denoted the G_1_ generation), with a similar F_1_-type genetic structure. On the basis of previous mapping studies, these G_1_ individuals will have genotypes of known haplotype composition, including phase of the QTN allele. By appropriate notation of marker and QTN alleles, these genotypes can be given the form: M_U_-Q--M_D_/m_U_-q--m_D,_ where M_U_ and M_D_ are markers defining the upstream (M_U_) and downstream (M_D_) (1-α) CI boundaries of the original QTL of width C M (see Figure 1). Q and q are alternative alleles at the QTN, and a large number of markers with alternative alleles (not shown) are found spanning the region between M_U_ and M_D_.

We now describe the construction of the TRP mapping population in detail. Based on Weller and Soller [26], we then derive the required size of the TRP mapping population to achieve target (1-α) CI about the QTN point location and compare to that required by standard F_2_, BC or AIL mapping populations for equivalent precision.

### (ii) The G_2_ population and the G_**2R**_ individuals

Depending on the reproductive biology of the species, the G_1_ parent individuals are selfed (TRP-F_2_ design) or backcrossed to one of the parental lines (TRP-BC design) to produce a G_2_ progeny population some of whom carry a recombinant chromosome in the target QTL. In what follows, we continue with the analysis of the TRP-F_2_ design. The TRP-BC design is a simpler application of the same principles and will be briefly described in Appendix I.

The G_2_ progeny are genotyped for the markers M_U_ and M_D_, and G_2R_ individuals, each carrying a single recombinant chromosome in the interval M_U_ to M_D_, are identified (see Figure 1). In principle, this would also uncover triple recombinants but these will be in vanishingly small frequency and can safely be ignored. Since there are two types of recombinant haplotypes (M_U_---m_D_, and m_U_---M_D_) and two types of parental haplotypes (M_U_---M_D_, and m_U_---m_D_) there will be four types of G_2R_ individuals, as shown in Table 1. In addition, a small proportion (= C^2^) of G_2R_ individuals carrying two independent recombinant chromosomes will also be produced. In practice, these individuals would not be included in the G_2R_ group, as the families they produce do not contain non-recombinant progeny needed to correct for polygenic family effects (see section xv for further details). To make up for this, it will be sufficient to increase the size of the G_2_ population by the proportion C^2^, e.g., by 4% if the original CI is 0.20 M. This will be considered as negligible and will not be taken into account in what follows.

**Table 1:** Composition of the entire G_3_ population^1^

**G**_**3**_ **Class**	**G**_**2R**_ **parent**			
	M_U_m_D_/M_U_M_D_	M_U_m_D_/m_U_m_D_	m_U_M_D_/M_U_M_D_	m_U_M_D_/m_U_m_D_
Class I	M_U_m_D_/M_U_m_D_	M_U_m_D_/M_U_m_D_	m_U_M_D_/m_U_M_D_	m_U_M_D_/m _U_M_D_
	1/16 d A	1/16 d B	1/16 -d C	1/16 -d D
Class II	M_U_m_D_/M_U_M_D_	M_U_m_D_/m_U_m_D_	m_U_M_D_/M_U_M_D_	m_U_M_D_/m_U_m_D_
	2/16 d E	2/16 h F	2/16 h G	2/16 -d H
Class III	M_U_M_D_/M_U_M_D_	m_U_m_D_/m_U_m_D_	M_U_M_D_/M_U_M_D_	m_U_m_D_/m_U_m_D_
	1/16 d NR	1/16 -d NR	1/16 d NR	1/16 -d NR

^1^Each cell represents a G_3_ progeny group according to Class and the G_2R_ parent, showing: marker genotype of the progeny group (above); proportion of the progeny group in the total G3 population (below-left); genotypic value of the progeny group (below-center); code designation (A to F) of the progeny group (below-right). Class I, homozygous recombinant progeny; Class II, heterozygous recombinant progeny; Class III, homozygous non-recombinant progeny; NR, non-recombinant progeny group not included in the G_3R_ mapping population

The G_2R_ individuals are genotyped for the markers spanning the region between M_U_ and M_D_, identifying the haplotype of the recombinant chromosome and the point of recombination. This information will allow some selection among the G_2R_ individuals chosen to produce the G_3_ generation to obtain a more evenly spaced distribution of recombination points across the original QTL. It also enables the complete genotypes of the G_3_ generation to be inferred from their G_2R_ parents, without requiring further genotyping of the G_3_ individuals (this is explained in the next section).

### (iii) Producing the G_**3R**_ mapping population and identifying a new point location for the QTN

Continuing with the TRP-F_2_ design, each parent G_2R_ individual will produce on selfing three classes of G_3_ progeny: Class I, Double recombinants in proportion 0.25, carrying two recombinant haplotypes; Class II, Single recombinants in proportion 0.50, carrying one recombinant and one non-recombinant (parental) haplotype; and Class III, Double non-recombinants in proportion 0.25, carrying two non-recombinant parental haplotypes (Table 1). The three G_3_ genotype classes within each family are identified by genotyping the G_3_ population for the pair of flanking markers (M_U_ and M_D_) that define the target region. The combined Class I and Class II recombinant progeny of all G_2R_ individuals form the G_3R_ mapping population. Thus, in contrast to the usual F_2_, BC or even AIL designs, in which the mapping population consists of only a small proportion of informative recombinant progeny for any target QTL, the TRP mapping population consists primarily of informative recombinant progeny, targeted to a specific QTL. The non-recombinant Class III G_3_ progeny group is not included in the G_3R_ mapping population. However, as will be shown later (section xv), it will contribute to evaluation and correction of family polygenic values.

Once G_3R_ recombinant status is determined, further genotyping is not required to obtain full G_3R_ genotypes, since (neglecting rare new recombination), the full genotype of any G_3R_ individual across the small target c interval, is determined by the genotype of the G_2R_ parent of that individual.

At some point along the way, either before or after genotyping, the G_3_ or G_3R_ population is phenotyped. A suitable t-test (or other appropriate test) is implemented in the G_3R_ population for each marker in the interval M_U_ to M_D_, with data pooled across all G_3R_ families. The marker with the most significant t-test is identified as the new point location of the QTN, denoted M_1_. Our goal is to determine the total G_3_ population size, N_TG3_, required to have the (1 − α) CI about M_1_ equal to c = C/s. We do this in two steps. basing our approach on Weller and Soller [26]. As shown in that paper, for any given mapping population, the (1-α) confidence level of a symmetrical interval of width c M, about the point location of a QTN is solely a function of: (i) α, (ii) the number, K, of recombinant chromosomes across that interval in the mapping population, and (iii) the standardized allele substitution effect, d, at the QTN. It follows, that for given α and d, K is a constant. Consequently, the required size, N_G3_, of a mapping population to deliver a (1-α) CI of width c will be the size of the population that will deliver K recombinant chromosomes across an interval of size c. In the first step, then, we calculate K for the given d and target α. N_G3_ will then depend solely on the proportion, R, of recombinant chromosomes in the mapping population such that, N_G3_ = K/R. In the second step, we note that since the new point location of the QTN in the original QTL is not known, the entire original (1-α)CI = C M, is considered as composed of s consecutive smaller sub-CI each of width c M. Each of these s sub-CI will require generating a G_3_ population of size N_G3_. Thus, N_TG3_, the total number of G_3_ progeny required to map the QTL to the sub-CI of width c, within its original QTL will be:

$$ {\mathsf{N}}_{\mathsf{TG3}}=\mathsf{s}{\mathsf{N}}_{\mathsf{G3}} $$

We now derive expressions for N_G3_, as a function of α and d for the TRP-F_2_-design.

### (iv) Required size of the G_3_ mapping population for given α and d

At this point it is convenient to shift our attention from Table 1, which gives the overall composition of the G_3_ population; to Table 2 which focuses our attention on the new point location of the QTN at M_1_, and its boundary marker, M_2_. With respect to these markers, the G_3_ population derived from the G_2R_ parents has the composition shown in Table 2. All told there are four G_2R_ family types and eight G_3R_ recombinant progeny groups, denoted A to H. For each G_3R_ recombinant group, Table 2 also shows the genotypic value, and the expected frequency in that fraction of the G_3_ population defining the new 95% CI = c about M_1_, calculated on the assumption that each of the four G_2R_ parental types contributes equally to the G_3_ population.

**Table 2:** **Composition of the fraction of the G**_**3**_ **population centered on the new QTN point location (M**_**1**_ **), and its boundary marker (M**_**2**_ **)** ^**1**^

**G**_**3**_ **Class**	**G**_**2R**_ **parent**			
	M_1_m_2_/M_1_M_2_	M_1_m_2_/m_1_m_2_	m_1_M_2_/M_1_M_2_	m_1_M_2_/m_1_m_2_
Class I	M_1_m_2_/M_1_m_2_	M_1_m_2_/M_1_m_2_	m_1_M_2_/m_1_M_2_	m_1_M_2_/m_1_M_2_
	1/16 d A	1/16 d B	1/16 -d C	1/16 -d D
Class II	M_1_m_2_/M_1_M_2_	M_1_m_2_/m_1_m_2_	m_1_M_2_/M_1_M_2_	m_1_M_2_/m_1_m_2_
	2/16 d E	2/16 h F	2/16 h G	2/16 -d H
Class III	M_1_M_2_/M_1_M_2_	m_1_m_2_/m_1_m_2_	M_1_M_2_/M_1_M_2_	m_1_m_2_/m_1_m_2_
	1/16 d NR	1/16 -d NR	1/16 d NR	1/16 -d NR

^1^ Each cell shows a G_3_ progeny group according to Class and the G_2R_ parent, showing: marker genotype of the progeny group (above); proportion of the progeny group in the G_3R_ population (below-left); genotypic value of the progeny group (below-center); code designation (A to F) of the progeny group (below-right). Class I, homozygous recombinant progeny; Class II, heterozygous recombinant progeny; Class III, homozygous non-recombinant progeny; NR, non-recombinant progeny group not included in the G_3_ mapping population.

Given the G_3R_ mapping population, with single marker-mapping the new point location of the QTN will be at the marker, M_1_, with the greatest difference between alternative marker genotypes, with marker M_2_ defining the one-side limit of the target **(1−**$\boldsymbol{\upalpha}$**)**CI. As shown by Weller and Soller [26] the probability that the CI of QTL location includes the marker M_2_ located at a remove of L M from M_1_, is equal to the probability of obtaining the value

$$ {\mathsf{Z}}_{\upalpha /\mathsf{2}}=\mathsf{D}/\mathsf{S}\mathsf{E}\left(\mathsf{D}\right), $$

where,

Z_α/2_ is the standard normal variable corresponding to a probability of α/2, D = E(M_1_) - E(M_2_), where E(M_1_) is the expected effect at M_1_ (QTN located at the marker), E(M_2_) is the expected QTN effect at M_2_ (located at a remove from the QTN) considering recombinant individuals only; and SE(D) is the standard error of D. Some thought will show that only recombinant individuals in the region M_1_ to M_2_ can contribute to a difference in expectation for M_1_ and M_2_, as non-recombinants have the same value at both markers.

Letting italics denote the mean genotypic value of the corresponding marker genotype group (including recombinant genotypes only), we have

1 $$ \mathrm{E}\left({\mathrm{M}}_1\right)={M}_1{M}_1\mathit{\hbox{--}}{m}_1{m}_1,\kern0.5em \mathrm{E}\left({\mathrm{M}}_2\right)={M}_2{M}_2\mathit{\hbox{--}}{m}_2{m}_2,\kern0.5em \mathrm{D}=\left({M}_1{M}_1\mathit{\hbox{--}}{m}_1{m}_1\right)\mathit{\hbox{--}}\left({M}_2{M}_2\mathit{\hbox{--}}{m}_2{m}_2\right) $$

From Table 2 it is apparent that each of the four marker genotype groups is composed of three recombinant marker groups. For example the marker genotype group M_2_M_2_ is composed of recombinant genotype groups C, D, and G of Table 2 with respective genotypes: m_1_M_2_/m_1_M_2_, m_1_M_2_/m_1_M_2_, and m_1_M_2_/M_1_M_2_; having genotypic values -d, -d, and h; frequencies 1/16, 1/16, and 2/16 of the G_3_ population; and relative frequencies 1/4, 1/4, and 1/2 within the M_2_M_2_ genotype. The mean genotypic value of the M_2_M_2_ genotype group, including recombinants only *(M*_*2*_*M*_*2*_*)* is the mean of the genotype groups C, D, and G, weighted by their relative frequencies in the M_2_M_2_ recombinant group, i.e. 1/4, 1/4 and 1/2, respectively.

On this basis, letting *A, B, C, D, E, F, G, H,* represent the genotypic values of the corresponding genotype groups, we have

$$ {\mathsf{M}}_{\mathsf{1}}{\mathsf{M}}_{\mathsf{1}}=\left(A/4\right)+\left(B/4\right)+\left(E/2\right) $$

$$ {\mathsf{m}}_{\mathsf{1}}{\mathsf{m}}_{\mathsf{1}}=\left(C/4\right)+\left(D/4\right)+\left(H/2\right) $$

$$ {\mathsf{M}}_{\mathsf{2}}{\mathsf{M}}_{\mathsf{2}}=\left(C/4\right)+\left(D/4\right)+\left(G/2\right) $$

$$ {\mathsf{m}}_{\mathsf{2}}{\mathsf{m}}_{\mathsf{2}}=\left(A/4\right)+\left(B/4\right)+\left(F/2\right) $$

Substituting in (1) and combining like terms, we obtain

$$ \mathrm{D}=\left(1/2\right)\left(A+B-C-D+E+F-G-H\right) $$

Substituting genotypic values of the recombinant groups we have

$$ \mathsf{D} = \left(\mathsf{1}/\mathsf{2}\right)\left(\mathsf{d}+\mathsf{d}+\mathsf{d}+\mathsf{d}+\mathsf{d}+\mathsf{h}\hbox{-} \mathsf{h} + \mathsf{d}\right)=\mathsf{3}\mathsf{d} $$

To calculate SE(D), we assume that variance within the G_3_ families is the same as variance within the F_2_ generation (set to 1.0 for standardization). This is conservative, as the variance within G_3_ families will generally be less than in an F_2_ population, depending on degree of homzoygosity in their G_2R_ parent. On this basis, we can write:

*σ*^2^_A_ = *σ*^2^_B_ = *σ*^2^_C_ = *σ*^2^_D_ = 1/(*N*_*G*3_/16) = 16/*N*_*G*3_ (as each of these genotype groups comprises 1/16 of the G_3_ mapping population of size N_G3_

*σ*^2^_E_ = *σ*^2^_F_ = *σ*^2^_G_ = *σ*^2^_H_ = 1/(*N*_*G*3_/8) = 8/*N*_*G*3_ (as each of these genotype groups comprises 1/8 of the entire G_3_ mapping population).

Then,

$$ \mathsf{S}{\mathsf{E}}^{\mathsf{2}}\left(\mathsf{D}\right)=\left(\mathsf{1}/\mathsf{4}\right)\Big[\left(\mathsf{4}\left(\mathsf{1}\mathsf{6}/{\mathsf{N}}_{\mathsf{G3}}\right)+\mathsf{4}\left(\mathsf{8}/{\mathsf{N}}_{\mathsf{G3}}\right)\right]=\left(\mathsf{1}\mathsf{6}/{\mathsf{N}}_{\mathsf{G3}} + \mathsf{8}/{\mathsf{N}}_{\mathsf{G3}}\right)=\mathsf{24}/{\mathsf{N}}_{\mathsf{G3}} $$

Substituting, we have

$$ {\mathsf{Z}}_{\upalpha /\mathsf{2}}=\mathsf{3}\mathsf{d}/{\left(\mathsf{24}/{\mathsf{N}}_{\mathsf{G3}}\right)}^{\mathsf{0.5}} $$

Solving for N_G3_, we have

$$ {\mathsf{N}}_{\mathsf{G3}}=\mathsf{2}\mathsf{4}{{\mathsf{Z}}_{\upalpha /\mathsf{2}}}^{\mathsf{2}}/\mathsf{9}{\mathsf{d}}^{\mathsf{2}}=\mathsf{2}.\mathsf{67}{{\mathsf{Z}}_{\upalpha /\mathsf{2}}}^{\mathsf{2}}/{\mathsf{d}}^{\mathsf{2}} $$

For example, if we set α = 0.05 and d = 0.2, we have Z_α/2_ = 1.96, and N_G3_ = 256.4

Note, however, that the interval between M_1_ and M_2_ defines only half of the CI of QTL location. Hence, to cover the entire CI would require twice this

$$ {\mathsf{N}}_{\mathsf{G3}}=\mathsf{5}.\mathsf{34}{{\mathsf{Z}}_{\upalpha /\mathsf{2}}}^{\mathsf{2}}/{\mathsf{d}}^{\mathsf{2}} $$

On these assumed values for α and d, the total G_3R_ population required for CI(0.95) = N_G3_ = 512.85 This number is constant for given d, and α, and does not depend on the target size of the confidence interval e.g., for α = 0.05 and d = 0.25, N_G3_ = 328.22. If N_G3_ is achieved across a small interval, then the (1-α) CI will be narrow; and if N_G3_ is achieved across a large interval, than the (1-α) CI will be wide.

Note, that in contrast to the usual F_2_ or similar mapping populations where the difference between alternative marker genotype groups is a function of proportion of recombination between marker and QTL, this is not the case for the TRP design, for which, since it is based on recombinant progeny only, the difference between alternative marker genotype groups is independent of proportion of recombination.

### (v) Required total G_3_ population size, N_TG3_

Since the location of the QTN in the original QTL is not known, the entire original CI_(1-α)_ = C M, is considered as composed of s consecutive smaller sub-CI_(1-α)_, each of width c M (the target CI_(1-α)_). Each of these s subintervals will require generating a G_3_ population of size N_G3_. Thus, for the example above (α = 0.05, d = 0.2) the total number of G_3_ progeny required to map the QTL to the sub-CI of size c = C/s within its original QTLR will be :

$$ {\mathsf{N}}_{\mathsf{TG3}}=\mathsf{s}{\mathsf{N}}_{\mathsf{G3}}=\mathsf{s}\mathsf{512}.\mathsf{85} $$

As noted, this number is constant for given s, d, and α, and does not depend on the target size c. Continuing with the same example (α = 0.05, d = 0.20), if we are reducing an original CI of C = 0.20 M to a target CI of 0.10 M (s = 2), then N_G3_ = 512.85 , and N_TG3_ = 2*512.85 = 1025.70 Of this total, 75% are included in the actual G_3R_ mapping population, and 25% are produced but are non-recombinant and not included in the mapping population.

### (vi) Required number of **G**_**2R**_ parent individuals

The number of G_2R_ individuals needed to produce the required number of G_3_ progeny for each sub-CI of size c, is:

$$ {\mathsf{N}}_{\mathsf{G2R}}={\mathsf{N}}_{\mathsf{G3}}/\mathrm{n} $$

where

n is the number of progeny that are produced by selfing each G_2R_ parent.

Continuing our example, and assuming n = 50, N_G2R_ = 512.85/50 = 10.26. It is important that N_G2R_ is large enough to provide a sufficient density of recombination points across the target QTL to define the sub-CI boundaries with a degree of precision (see simulation for further details).

### (vii) Required **G**_**2**_ population size

By definition, when c is small the proportion of G_1_ gametes carrying a recombinant chromosome across the interval c M, will be c. In the G_2_ generation produced from these gametes, a proportion C^2^ will carry two recombinant chromosomes and are excluded from the G_3R_ mapping population as explained above. As C^2^ is generally small it is conveniently neglected in the calculations. In the remaining G_3R_ population, all recombinants across the region c, will be present in proportion 2c(1-c), as heterozygotes carrying a recombinant chromosome together with a parental haplotype. Thus, as a close approximation, a bit on the high side, we have

${\mathsf{N}}_{\mathsf{G2}\mathsf{R}}=\mathsf{2}\mathsf{c}\left(\mathsf{1}\hbox{-} \mathsf{c}\right){\mathsf{N}}_{\mathsf{G2}}$ and solving for N_G2_ we have

$$ {\mathsf{N}}_{\mathsf{G2}}={\mathsf{N}}_{\mathsf{G2}\mathsf{R}}/\mathsf{2}\mathsf{c}\left(\mathsf{1}\hbox{-} \mathsf{c}\right) $$

Noting that N_G2R_ = N_G3_/n and N_G2_ = N_G2R_/2c(1-c), we can solve for N_G2_ as a function of N_G3_

$$ {\mathsf{N}}_{\mathsf{G2}}={\mathsf{N}}_{\mathsf{G3}}/\mathsf{2}\mathrm{n}\mathsf{c}\left(\mathsf{1}\hbox{-} \mathsf{c}\right) $$

This is convenient as N_G3_ is the basic number determining all the remaining numbers.

The same total of G_2_ individuals needed to yield N_G2R_ recombinant individuals for one sub-CI of size c M, simultaneously produces N_G2R_ recombinant individuals for each sub-CI of size c M across the entire genome. Hence, the same G_2_ population of size N_G2_ can provide N_G2R_ individuals for all sub-CI of the designated QTL (and indeed, as noted previously, for any other QTL mapped in the original F_2_ population).

Thus, for our example, C = 0.2, c = 0.1, n = 50, N_G3_ = 512.75 , so that

$$ {\mathsf{N}}_{\mathsf{G2}}=512.85/2*0.1*0.90*50=56.98 $$

That is, a G_2_ population of size 56.98 individuals (113.97 chromosomes), will contain 10.26 G_2R_ recombinants for each target interval of 0.1 M in an initial CI of size 0.2M.

### (viii) Required **G**_**1**_ population size

The number of G_1_ individuals required to produce the G_2_ population will obviously equal

$$ {\mathsf{N}}_{\mathsf{G1}}={\mathsf{N}}_{\mathsf{G2}}/\mathrm{n}={\mathsf{N}}_{\mathsf{G3}}/\left(\mathsf{2}\mathrm{n}\mathsf{c}\left(\mathsf{1}\hbox{-} \mathsf{c}\right)\right)/\mathrm{n}={\mathsf{N}}_{\mathsf{G3}}/\mathsf{2}{\mathrm{n}}^{\mathsf{2}}\mathsf{c}\left(\mathsf{1}\hbox{-} \mathsf{c}\right) $$

For our example, N_G1_ = 512.85/450 = 1.14, so that 2 or 3 G_1_ individuals should suffice.

### (ix) Total progeny required across the G_1_**,** G_**2**_ and G_**3**_ generations (N_TRP_)

Combining expressions for N_G1_, N_G2_ and N_TG3_, as function of N_G3_, we have

$$ {\mathsf{N}}_{\mathsf{TRP}}={\mathsf{N}}_{\mathsf{G3}}/\mathsf{2}{\mathrm{n}}^{\mathsf{2}}\mathsf{c}\left(\mathsf{1}\hbox{-} \mathsf{c}\right)+{\mathsf{N}}_{\mathsf{G3}}/\mathsf{2}\mathrm{n}\mathsf{c}\left(\mathsf{1}\hbox{-} \mathsf{c}\right)+\mathsf{s}{\mathsf{N}}_{\mathsf{G3}} $$

$$ = {\mathsf{N}}_{\mathsf{G3}}\left(\mathsf{1}/\mathsf{2}{\mathrm{n}}^{\mathsf{2}}\mathsf{c}\left(\mathsf{1}\hbox{-} \mathsf{c}\right)+\mathsf{1}/\mathsf{2}\mathrm{n}\mathsf{c}\left(\mathsf{1}\hbox{-} \mathsf{c}\right)+\mathsf{s}\right) $$

For our example, N_G3_ = 512.85, n = 50, c = 0.1, s = 2

$$ {\mathrm{N}}_{\mathrm{TRP}}=1093.91 $$

The contribution of N_G1_ is always negligible, and can safely be ignored. For the present example with relatively large c, the contribution of N_G2_ is small, but when c is small, this is not the case, and N_G2_ makes an appreciable contribution to N_TRP_.

To reduce the same 20 cM original QTL to 2 cM (s = 10), would require 5,196.71 individuals. All the increase will come from the increased G_3_ population; G_1_ and G_2_ remain the same.

### (x) Total required genotyping and phenotyping

All individuals of the G_2_ generation are genotyped for the pair of markers flanking the original QTL to identify the G_2R_ individuals. These, making up 2c(1-c) of the G_2_ generation, are genotyped for k internal markers to locate the QTN more precisely within the original QTL. Based on the simulation results, about k = 30 to 50 internal markers should be genotyped in the G_2R_ individuals to extract maximum mapping information from a given G_3_ population. Finally, the entire G_3_ population is genotyped for a single marker, to identify their genotype (Class I, Class II or Class III in Tables 1 and 2) with respect to the haplotype transmitted from their G_2R_ parent. Thus, total genotyping data points (g.d.p.) will be

$$ \mathsf{Total}\kern0.5em \mathsf{g}.\mathsf{d}.\mathsf{p}.=\mathsf{2}{\mathsf{N}}_{\mathsf{G2}}+\mathsf{2}\mathsf{c}\left(\mathsf{1}\hbox{-} \mathsf{c}\right)\mathsf{k}{\mathsf{N}}_{\mathsf{G2}}+{\mathsf{N}}_{\mathsf{TG3}} $$

For example, from the combination C = 0.02, s = 4, c = 0.005 we have N_TG3_ = 7,591, N_G2_ = 3,814. Then, taking k = 50, we have Total g.d.p. = 15,131: a remarkably low figure considering today’s genotyping capacities and costs.

**Table 3:** **Population size for TRP-F2 design by width of initial QTL and target QTL , polygenic effect absent** ^**1**^

**C**	**s**	**c**	**N**_**TG3**_	**N**_**G2**_	**N**_**TRP**_	**N**_**F2**_	**N**_**TRP**_ **/N**_**F2**_
0.20	2	0.100	1026	57	1083	3750	0.289
0.20	4	0.050	2051	108	2159	7500	0.288
0.20	10	0.020	5129	261	5390	18750	0.287
0.10	2	0.050	1026	108	1134	7500	0.151
0.10	10	0.010	5129	517	5646	37500	0.151
0.05	2	0.025	1026	211	1237	15000	0.082
0.05	5	0.010	2564	517	3081	37500	0.082
0.05	10	0.005	5129	1029	6158	75000	0.082
0.02	2	0.010	1026	517	1543	37500	0.041
0.02	4	0.005	2051	1046	3097	75000	0.041
0.02	10	0.002	5129	2564	7693	187500	0.041

^1^Abbreviations: C, the original QTL in Morgans; s, the reduction factor; c, target QTL in Morgans; N_TG3_, total size of G_3_ population; N_G2_, total size of G_2_ population. N_TRP_, total number required across G_2_ and G_3_ populations; N_F2_, population size for equivalent QTL width using an F_2_ design. Assumptions: polygenic effect absent; standardized allele substitution effect, d = 0.2; reproductive potential of the G_2_ generation, n = 50; and confidence level set at (1-α) = 95%

With respect to phenotyping, the entire G_3_ population is phenotyped, but there is no need to phenotype the G_2_ generation. If phenotyping costs are a major consideration, some form of selective phenotyping [18,19] may be useful to reduce phenotyping data points at the expense of an increase in total population size and a negligible increase in genotyping costs

### (xi) Total progeny required for an F_2_ mapping population to achieve equivalent map resolution:

Following Weller and Soller [26], population size required by an F_2_ population to achieve 95% CI = c M is:

$$ {\mathsf{N}}_{\mathsf{F2}}=\mathsf{15}/{\mathsf{d}}^{\mathsf{2}}\mathsf{c} $$

For our example, d = 0.2, c = 0.1 we have

$$ {\mathsf{N}}_{\mathsf{F2}}=\mathsf{3750} $$

Noting that in our example N_G3_ = 5.34(Z_α/2_^2^)/d^2^, the ratio of N_TRP_ to N_F2_ is given by

$$ {\mathsf{N}}_{\mathsf{TRP}}/{\mathsf{N}}_{\mathsf{F2}}=\mathsf{5}.\mathsf{34}\left(\mathsf{1}.\mathsf{9}{\mathsf{6}}^{\mathsf{2}}\right)/{\mathsf{d}}^{\mathsf{2}}\Big)\left(\mathsf{1}/\mathsf{2}\mathrm{n}\mathsf{c}\left(\mathsf{1}\hbox{-} \mathsf{c}\right)+\mathsf{s}\right)/{\mathsf{N}}_{\mathsf{F2}} $$

$$ =\left(\mathsf{20.5}/{\mathsf{d}}^{\mathsf{2}}\right)\left(\mathsf{1}/\mathsf{2}\mathrm{n}\mathsf{c}\left(\mathsf{1}\hbox{-} \mathsf{c}\right)+\mathsf{s}\right)/\mathsf{15}/\left({\mathsf{d}}^{\mathsf{2}}\mathsf{c}\right) $$

$$ =\mathsf{20}.\mathsf{5}\left(\mathsf{1}/\mathsf{2}\mathrm{n}\mathsf{c}\left(\mathsf{1}\hbox{-} \mathsf{c}\right)+\mathsf{s}\right)/\mathsf{15}/\mathsf{c} $$

$$ =\mathsf{43}.\mathsf{27}/\mathsf{150}=\mathsf{0}.\mathsf{288}\kern0.5em \mathsf{f}\mathsf{o}\mathsf{r}\kern0.5em \mathsf{c}=\mathsf{0}.\mathsf{1},\mathsf{a}\mathrm{n}\mathsf{d}\kern0.5em \mathsf{0}.\mathsf{0288}\kern0.5em \mathsf{f}\mathsf{o}\mathsf{r}\kern0.5em \mathsf{c}=\mathsf{0}.\mathsf{01} $$

Examination of the final expression for N_TRP_/N_F2_ shows that it is a function of n, c, and s only, and is not affected by α, nor, even more remarkably, by d.

### (xii) Genotyping and phenotyping the F_2_

All N_F2_ individuals of the F_2_ mapping population are genotyped for the two markers flanking the original QTL of width C. This will identify 2C(1-C) F_2R_ individuals carrying a recombinant chromosome in the target region. These are then genotyped for all k internal markers. Thus, Total g.d.p. = 2N_F2_ + 2C(1-C)kN_F2_. For the example we used for the TRP design (C = 0.02, s = 4, c = 0.005), we have N_F2_ = 75,000, giving Total g.d.p. = 297,000; 20-fold more than required by the TRP design.

Ordinarily, all N_F2_ individuals would be phenotyped. However, if genotyping precedes phenotyping there is opportunity to greatly reduce phenotyping numbers by phenotyping the recombinant F_2_ individuals only. Similarly, if phenotyping precedes genotyping it may be possible to reduce genotyping numbers by use of selective genotyping [27] or even selective DNA pooling [28,29].

### (xiii) Mapping requirements in other TRP designs

Mapping requirements in an TRP-AIL are the same as in an TRP-F_2_, except that the mapping population sizes are less by a factor of 0.5t, where t is the generation number of the AIL. Mapping population size requirements in a TRP-BC depend strongly on degree of dominance at the QTL (h), and range from twice to half that of an TRP-F_2_, depending on whether h = 0 or h = 1, respectively. For the case h = 0, we expect the TRP-BC to require about twice as many data points as a TRP-F2 for equivalent power, and this is indeed what is found (Appendix I).

### (xiv) Polygenic variance component of SE(D)

To this point we have calculated SE(D) on the assumption that the expected value of the G_3_ marker genotype group within families is μ + d , μ-d or μ + h, depending on the genotype at the QTL. The underlying assumption being that μ is the same for all families and marker groups within families. This is true for a standard F_2_ population, where all F_1_ parent individuals have the same polygenic value.

However, in the TRP-F_2_ design, each G_3_ family is generated by selfing from a different G_2R_ individual. These will differ in polygenic value with standardized polygenic variance between the G_3_ families equal to the heritability (h^2^) of the trait in the G_2_ generation plus a small dominance component [30]. This has a strong effect on the SE(D). In the absence of a polygenic effect, and using the previous notation, we have

$$ \mathsf{D}=\left(\mathsf{1}/\mathsf{2}\right)\left(\mathsf{A}+\mathsf{B}-\mathsf{C}-\mathsf{D}+\mathsf{E}+\mathsf{F}-\mathsf{G}-\mathsf{H}\right) $$

Four families types are represented (Table 2). Groups A and E are derived from Family type 1; Groups B and F from Family type 2; groups C and G from Family type 3 and groups D and H from Family type 4. Then, in the presence of a polygenic effect, to each of the groups, in addition to the expected genotypic value, we need to add a polygenic value taken from a normal distribution with mean 0 and variance h^2^.

Letting *P*_*1*_*, P*_*2*_*, P*_*3*_*, P*_*4*_ be the polygenic effects of Families 1, 2, 3, 4 respectively, we have:

$$ \mathsf{D}\hbox{'}=\left(\mathsf{1}/\mathsf{2}\right)\left(\mathsf{A}+{\mathsf{P}}_{\mathsf{1}}+\mathsf{B}+{\mathsf{P}}_{\mathsf{2}}-\mathsf{C}-{\mathsf{P}}_{\mathsf{3}}-\mathsf{D}-{\mathsf{P}}_{\mathsf{4}}+\mathsf{E}+{\mathsf{P}}_{\mathsf{1}}+\mathsf{F}+{\mathsf{P}}_{\mathsf{2}}-\mathsf{G}-{\mathsf{P}}_{\mathsf{3}}-\mathsf{H}-{\mathsf{P}}_{\mathsf{4}}\right) $$

$$ =\left(1/2\right)\left(A+B-C-D+E+F-G-H+2{P}_1+2{P}_2\hbox{--} 2{P}_3\hbox{--} 2{P}_4\right) $$

As before

$$ {\sigma^{\mathsf{2}}}_{\mathsf{A}}={\sigma^{\mathsf{2}}}_{\mathsf{B}}={\sigma^2}_{\mathsf{C}}={\sigma^2}_{\mathsf{D}}=\mathsf{1}/\left({\mathsf{N}}_{\mathsf{G3}}/\mathsf{16}\right)=\mathsf{1}\mathsf{6}/{\mathsf{N}}_{\mathsf{G3}} $$

$$ {\sigma^{\mathsf{2}}}_{\mathsf{E}}={\sigma^{\mathsf{2}}}_{\mathsf{H}}=\mathsf{1}/\left({\mathsf{N}}_{\mathsf{TG3}}/\mathsf{8}\right)=\mathsf{8}/{\mathsf{N}}_{\mathsf{G3}} $$

*σ*^2^(2*P*_1_) = *σ*^2^(2*P*_2_) = *σ*^2^(2*P*_3_) = *σ*^2^(2*P*_4_) = 4*h*^2^/*k*, where k is the average number of replicate families of each type.

Then

$$ \mathsf{S}{\mathsf{E}}^{\mathsf{2}}\left(\mathsf{D}\hbox{'}\right)=\left(\mathsf{1}/\mathsf{4}\right)\left(\mathsf{4}*\mathsf{16}/{\mathsf{N}}_{\mathsf{G3}}+\mathsf{4}*\mathsf{8}/{\mathsf{N}}_{\mathsf{G3}}+\mathsf{4}*\mathsf{4}{\mathsf{h}}^{\mathsf{2}}/\mathsf{k}\right) $$

$$ =\left(\mathsf{16}/{\mathsf{N}}_{\mathsf{G3}}+\mathsf{8}/{\mathsf{N}}_{\mathsf{G3}}+\mathsf{4}{\mathsf{h}}^{\mathsf{2}}/\mathsf{k}\right)=\mathsf{24}/{\mathsf{N}}_{\mathsf{G3}}+\mathsf{4}{\mathsf{h}}^{\mathsf{2}}/\mathsf{k} $$

The polygenic component has a powerful effect. For example, if N_G3_ = 1000, n = 50, then k = (1000/50)/4 = 5, Then if h^2^ = 0.25, we have SE^2^(D) = 24/1000 = 0.024 in absence of a polygenic effect; while the polygenic effect will add 4*0.25/5 = 0.20, to give SE^2^(D’) = 0.224. For SE^2^(D’) to equal 0.024 in the presence of a polygenic effect, we would need to increase N_G3_ tenfold, from 1000 to 10,000, giving k = 50, and SE^2^(D’) = 24/10,000 + 4h^2^/50 = 0.0224.

### (xv) Using non-recombinant progeny to correct for polygenic effects

To deal with polygenic effects in a more effective manner, we propose to use the non-recombinant group of each family as an estimate of the family polygenic effect, and express the recombinant groups as deviations from the non-recombinant group. Examination of Table 2 shows that there are two classes of non-recombinant groups. For Families 1 and 3, the non-recombinant groups have genotype: M_1_QM_2_/M_1_QM_2_, with genotype values + d; for Families 2 and 4, the non-recombinant groups have genotype m_1_qm_2_/ m_1_qm_2_ , with genotype values –d. Thus, to bring all non-recombinant families to the same expectation, we need to estimate d from the previous mapping experiment that defined the original QTL, and correct Families 2 and 4 for the effect of the QTN by adding 2d to the non-recombinant groups of these families. When this is done, the variation among non-recombinant groups of the different families, will be due to polygenic variation alone. Thus, the mean of the non-recombinant group will represent the polygenic effect of the family and will be common to all genotype groups within the family. Consequently, the expected deviation of the recombinant groups from the non-recombinant group mean will be due to genotype at the target QTL only, and not due to polygenic effects.

Although the expected polygenic effect of the non-recombinant group and of the recombinant groups is the same, the mean of a non-recombinant group will have a sampling variance, depending on the number of individuals in the group. Since proportion of total family in the non-recombinant groups is 1/16, this will equal 16/N_G3_. Note, in this case, k is not relevant as it does not make any difference how many subfamilies are within a given family, sampling variance of the non-recombinant groups will depend on the total population size only, and not on how it is divided among replicate families.

If we let *Q*_*1*_*, Q*_*2*_*, Q*_*3*_*, Q*_*4*_, be the sampling deviation of the non-recombinant group from expectation, we have

$$ \mathsf{D}=\left(\mathsf{1}/\mathsf{2}\right)\left(\mathsf{A}+{\mathsf{Q}}_{\mathsf{1}}+\mathsf{B}+{\mathsf{Q}}_{\mathsf{2}}-\mathsf{C}-{\mathsf{Q}}_{\mathsf{3}}-\mathsf{D}-{\mathsf{Q}}_{\mathsf{4}}+\mathsf{E}+{\mathsf{Q}}_{\mathsf{1}}+\mathsf{F}+{\mathsf{Q}}_{\mathsf{2}}-\mathsf{G}-{\mathsf{Q}}_{\mathsf{3}}-\mathsf{H}-{\mathsf{Q}}_{\mathsf{4}}\right) $$

$$ =\left(\mathsf{1}/\mathsf{2}\right)\left(\mathsf{A}+\mathsf{B}-\mathsf{C}-\mathsf{D}+\mathsf{E}+\mathsf{F}-\mathsf{G}-\mathsf{H}+\mathsf{2}{\mathsf{Q}}_{\mathsf{1}}+\mathsf{2}{\mathsf{Q}}_{\mathsf{2}}\hbox{--} \mathsf{2}{\mathsf{Q}}_{\mathsf{3}}\hbox{--} \mathsf{2}{\mathsf{Q}}_{\mathsf{4}}\right) $$

Where, as before

$$ {\sigma^2}_{\mathrm{A}}={\sigma^2}_{\mathrm{B}}={\sigma^2}_{\mathrm{C}}={\sigma^2}_{\mathrm{D}}=\mathsf{1}/\left({\mathsf{N}}_{\mathsf{G3}}/\mathsf{16}\right)=\mathsf{1}\mathsf{6}/{\mathsf{N}}_{\mathsf{G3}} $$

$$ {\sigma^2}_{\mathrm{E}}={\sigma^2}_{\mathrm{H}}=1/\left({\mathsf{N}}_{\mathsf{G3}}/\mathsf{8}\right)=\mathsf{8}/{\mathsf{N}}_{\mathsf{G3}} $$

$$ {\sigma}^2\left(2{Q}_1\right)={\sigma}^2\left(2{Q}_2\right)={\sigma}^2\left(2{Q}_3\right)={\sigma}^2\left(2{Q}_4\right)=4*16/{\mathsf{N}}_{\mathsf{G3}}, $$

Then SE^2^(D) = (1/4)(4*16/N_G3_ + 4*8/N_G3_ + 4*4*16/N_G3_) = (16/N_G3_ + 8/N_G3_ + 64/N_G3_) = 88/N_G3_

Substituting and solving for N we have

$$ {\mathsf{Z}}_{\upalpha /\mathsf{2}}=\mathsf{3}\mathsf{d}/{\left(\mathsf{88}/{\mathsf{N}}_{\mathsf{G3}}\right)}^{\mathsf{0.5}} $$

And solving for N_G3_, we have

N_G3_ = 88Z_α/2_^2^/9d^2^ = 9.88Z_α/2_^2^/d^2^ for one side of the CI, and twice this, 19.76Z_α/2_^2^/d^2^ for two-sided CI.

If we set α = 0.05, d = 0.2 as in our example, we have Z_α/2_ = 1.96, and total G_3_ population required for a two-side CI, N_G3_ = 1897.75. This can be compared to the corresponding value N_G3_ = 512.85 in the absence of polygenic effects. Since all other numbers in the analysis are functions of N_G3_, the presence of polygenic effects of magnitude h^2^ = 0.25, increases required populations sizes 3.7-fold all down the line. Relative to a standard F_2_ design, TRP-F_2_ population sizes for equivalent power as a proportion of the required F_2_ population would increase from 0.288 for c = 0.1, s = 2 in the absence of polygenic effects, to 1.068 in their presence, i.e., no savings. For c = 0.01, s = 2, however, relative savings would be 0.152, which is still appreciable.

### Simulation

The above calculations assume a saturated marker map, and saturation of the QTL by points of recombination. A simulation study was carried out to study the effect of marker spacing, and number of G_2R_ families (i.e., number of randomly spaced points of recombination per target CI) on the Standard Error of QTN map location (SEQTN). For simplicity we simulated a TRP-BC (backcross) design, and assumed absence of polygenic effects.

As before, we assume that conventional QTL mapping in a BC, F_2_ or AIL population has detected a QTL of interest with a 95% confidence interval (CI) defined by a pair of flanking upstream and downstream markers, denoted M_U_ and M_D_, respectively. A very large set of evenly spaced ordered makers, denoted M_1_ to M_k_ spanning the interval M_U_ to M_D_ is available, and haplotypes of the parental lines (denoted the G_1_ generation) with respect to these markers are known. In the proposed scheme, the original or a new mapping population (the G_2_ generation) is genotyped for the M_U_ - M_D_ marker pair, and G_2R_ individuals carrying recombinant chromosomes across this region (i.e., m_U_-M_D_ and M_U_–m_D_) are identified. These are backcrossed to one of the original founder lines, and a large simulated BC progeny population (the G_3_ generation) is formed, consisting of a number of G_2R_ families, and genotyped for the marker set spanning the QTL. A QTN is simulated at a specific location in the QTL, and the marker showing the largest difference between alternative marker genotypes is identified as the estimated QTN location. The distance between the estimated QTN location and the simulated location was determined in units of the initial QTL, and the accuracy of map location was evaluated as the SEQTN. For each combination of parameters, 1000 Monte Carlo simulations were run.

To investigate the effect of population size (N), number of internal markers (k) and QTN location (L) on SEQTN we set: N = 1000, 3000, 5000, 7000, 9000, 11000; k = 2, 5, 10, 23, 30, 40, 50 (k does not include the flanking M_U_ and M_D_ markers, e. g., k = 2 represents a total of 4 markers, the two flanking markers and two internal markers); allele substitution effect in standardized units, d = 0.2; number of G_2R_ families, F = 25; QTN location, L = 0.57, 0.77, again taking the width of the CI = 1.0 as the unit of measure. Thus, L = 0.57 is a bit distal to the center of the QTL, and L = 77 is slightly distal to the three-quarter mark. QTN positions and the k = 23 marker number were chosen to ensure that in no instance did marker position and simulated QTN position coincide.

To investigate effect of number of families within a QTL on SEQTN we set N = 3000, 11000; F = 500, 250, 100, 50, 25, 10; L = 0.57; k and d as before.

The simulation results were obtained as SEQTN, while the results of the deterministic analysis are presented as the population size required to achieve a given factor of QTL reduction, s. To compare the two approaches we converted both of their outputs to the achieved reduction factor, s. For SEQTN, we assumed that the new confidence interval of QTN location would equal 4*SEQTN (i.e., two standard deviations to each side). Thus, taking the original QTL = 1.0, and the new QTL = 4(SEQTN), the reduction factor would be s = 1/4(SEQTN). For the deterministic analysis, we have N_TG3_ = sN_G3_, so that s = N_TG3_/N_G3_.

For example, in the simulation, at N = 7000 (L = 0.57), we obtain SEQTN = 0.0575. Then s = 1/4(SEQTN) = 4.35. In the deterministic analysis of the TRP-BC design, N_G3_ = 1536.64 (see Results). Then, s = 7000/1536.64 = 4.56.

**Table 4:** **Population size for TRP-F2 design, polygenic effect present** ^**1**^

**C**	**s**	**c**	**N**_**TG3**_	**N**_**G2**_	**N**_**TRP**_	**N**_**F2**_	**N**_**TRP**_ **/N**_**F2**_
0.20	2	0.100	3796	211	4007	3750	1.068
0.20	4	0.050	7591	400	7991	7500	1.065
0.20	10	0.020	18978	968	19946	18750	1.064
0.10	2	0.050	3796	400	4196	7500	0.559
0.10	10	0.010	18978	1917	20895	37500	0.557
0.05	2	0.025	3796	779	4575	15000	0.305
0.05	5	0.010	9489	1917	11406	37500	0.304
0.05	10	0.005	18978	3814	22793	75000	0.304
0.02	2	0.010	3796	1917	5713	37500	0.152
0.02	4	0.005	7591	3814	11406	75000	0.152
0.02	10	0.002	18978	9508	28486	187500	0.152

^1^Abbreviations: C, the original QTL in Morgans; s, the reduction factor; c, target QTL in Morgans; N_TG3_, total size of G_3_ population; N_G2_, total size of G_2_ population. N_TRP_, total number required across G_2_ and G_3_ populations; N_F2_, population size for equivalent QTL width using an F_2_ design. Assumptions: polygenic effect present; standardized allele substitution effect, d = 0.2; reproductive potential of the G_2_ generation, n = 50; and confidence level set at (1-α) = 95%

## Results

As shown in Methods, the basic parameter determining the required numbers of G_3_ progeny and G_2_ parents under the TRP design, is N_G3_, the number of G_3_ individuals needed to define a single (1-α) CI for given allele substitution effect, d. For a 95% CI in the absence of polygenic effects, we derived the expression N_G3_ = 5.34z_α/2_^2^/d^2^. Taking 95% CI as the standard, z_α/2_ = 1.96, and folding this into the constant, we have N_G3_ = 20.51/d^2^. This shows starkly that N_G3_ will be very sensitive to the allele substitution effect, d, e.g., in absence of polygenic effects, N_G3_ = 2051.0, 512.75, and 227.9 for d = 0.1, 0.2 and 0.3, respectively. We stress again that these numbers are independent of the size of the original QTL (C) or of the target sub-QTL (c), or the reduction factor (s). It makes no difference if the reduction is from 20 cM to 2 cM, or from 2 cM to 0.2 cM, N_G3_ will be the same. The same holds true in the presence of polygenic effects, except that in this case, the basic expression for N_G3_ = 75.91/d^2^

The total G_3_ population, N_TG3_ required for mapping under TRP, depends solely on N_G3_ and s, the desired reduction-factor in QTL size. A five-fold reduction means that the original QTL will be divided into 5 sub-QTL. This will require the same number, N_G3_ of individuals for each of the sub-QTL. Consequently, N_TG3_ = sN_G3_, where s is the number of sub-QTL into which we divide our original QTL.

The total G_2_ population required to produce the G_3_ population in absence of polygenic effects, depends on N_G3_, and on the reproductive potential (n) and target interval (c) as follows, N_G2_ = N_G3_/2nc(1-c). Thus, it stands in proportion to 1/2nc(1-c). Assuming, n = 50, 1/2nc(1-c) will be quite small when c is large, e.g., for C = 0.2 M, c = 0.1 M, 1/2nc(1-c) = 0.111 and N_G2_ = 56.98 indeed very small (5%) relative to N_TG3_ = 1026 for this case (Table 3). However, 1/2nc(1-c) will assume larger values when c is small; e.g., for C = 0.1, c = 0.01, 1/2nc(1-c) = 0.99, N_G2_ = 507.72 and N_TG3_ = 5129 (10%); and for C = 0.2. c = 0.002, 1/2nc(1-c) = 5.01, and N_G2_ = 2569 and N_TG3_ = 5129, (50%).

**Table 5:** **Standard error of estimated QTN location by simulation, as a function of G**_**3**_ **population size (N)** ^**1**^

**N**	**k = 2**	**5**	**10**	**23**	**30**	**40**	**50**	**sSIM**	**sDET**
L = 0.57									
1000	2.0700	0.7220	0.3340	0.2030	0.1890	0.1820	0.1820	1.37	0.65
3000	0.7640	0.1180	0.0974	0.0959	0.0936	0.0921	0.0926	2.70	1.95
5000	0.4200	0.0993	0.0758	0.0734	0.0711	0.0728	0.0712	3.51	3.25
7000	0.1270	0.0905	0.0659	0.0580	0.0569	0.0581	0.0575	4.35	4.56
9000	0.1140	0.0895	0.0587	0.0500	0.0493	0.0491	0.0485	5.15	5.86
11000	0.1200	0.0859	0.0575	0.0499	0.0494	0.0472	0.0453	5.52	7.16
L = 0.77									
1000	1.8100	0.3700	0.1890	0.1730	0.1680	0.1710	0.1700	1.47	0.65
3000	0.4950	0.1090	0.0964	0.0909	0.0894	0.0900	0.0883	2.83	1.95
5000	0.1380	0.0943	0.0723	0.0691	0.0680	0.0681	0.0672	3.72	3.25
7000	0.1330	0.0864	0.0659	0.0570	0.0551	0.0553	0.0559	4.47	4.56
9000	0.1310	0.0828	0.0597	0.0499	0.0489	0.0492	0.0494	5.06	5.86
11000	0.1280	0.0844	0.0567	0.0490	0.0461	0.0458	0.0460	5.43	7.16

^1^Abbreviations and assumptions: Standard error of estimated QTN location (SEQTN) by simulation, as a function of G3 population size (N), family size, (I); location of the QTN within its confidence interval (L) and number of markers spanning the QTL (k). sSIM, reduction factor according to the simulation analysis; sDET, reduction factor according to the deterministic analysis. Standardized allele substitution effect at the QTN = 0.2; number of G_2R_ families, F = 25

Table 3 shows population size required for the G_2_ and G_3_ stages of the TRP design and total numbers across both generations as a function of the width of the original QTL (C), the target sub-QTL (c), and the reduction factor, s, on the assumptions of no polygenic effect, d = 0.2, n = 50 and α = 0.05. Population size for TRP designs relative to F_2_ designs range from 0.289 for initial CI of 0.2 M to 0.041 for initial CI of 0.02 M. Thus, the clear conclusion is that the TRP design will be most useful when the initial CI of the QTL has been brought to fairly high resolution already, and the desired step is ultra-high resolution. Within a given initial CI, the relative effectiveness of the TRP designs compared to the F_2_ designs does not depend on the target CI. Of course, the actual required numbers vary considerably, as seen in Table 3. But the relative numbers required remain the same.

The table shows clearly that N_TG3_ varies directly with and depends solely on the reduction factor, s; while N_G2_ varies inversely with the original QTL and target sub-QTL size, and directly with the reduction factor.

In contrast to the required number of G_3_ and G_2R_ individuals, which is a function only of the reduction factor, the total number of G_2_ individuals depends strongly on the target CI. This is due to the fact that it requires, for example, ten times as many total G_2_ individuals to uncover a given number of G_2R_ individuals in a region of 0.02 M, as compared to a region of 0.2 M. Thus, the number of G_2_ individuals ranges from 5% of the number of G_2_ individuals when initial CI is 0.2 M; to ten times this, or 50% when initial CI is 0.02 M.

In the absence of polygenic effects, the TRP delivers major reductions in mapping population size relative to a standard F_2_ design, depending on the initial QTL size. This is particularly evident for very high resolution, c = 0.01M or 0.002M, where the TRP ostensibly delivers ultra-high resolution at very acceptable population sizes, while the F_2_ design requires very high numbers for mapping at this resolution. Sadly, this fine performance is markedly reduced when polygenic effects are taken into account (Table 4). In this case, the relative effectiveness of the TRP design depends strongly on the initial QTL start width. Indeed, when start point is at C = 0.2, the TRP design required a bit larger population size than an F_2_. When the initial interval is small (e.g., 0.05 or 0.02 M), TRP requires only about 15% the population size of an F2 for equivalent mapping precision. In this case, TRP can close the gap to 0.01 M with manageable numbers (11,406 and 5,713, for C = 0.05 and C = 0.02, respectively).

For species with short generation interval (annual plants, mice) a two- stage TRP can start with a large initial CI, say C = 0.2M, and yet reach c = 0.01 in two steps, with acceptable total population numbers. For example, starting with C = 0.2 M, first stage might reduce four-fold to 0.05 M requiring N_TRP_ = 7991; second stage would reduce four-fold again to 0.0125 M requiring N_TRP_ = 7991, total 15,982, spread fairly equally over two years; while F_2_ would require 30,000.

### The number of recombination points per sub-QTL

The number of G_2R_ individuals needed to produce the required number of G_3_ progeny for each sub-CI of size c, is:

$$ {\mathsf{N}}_{\mathsf{G2R}}={\mathsf{N}}_{\mathsf{G3}}/\mathrm{n} $$

where

n is the number of progeny that are produced by selfing each G_2R_ parent.

Continuing our example, and assuming n = 50, then N_G2R_ = 512.75/50 = 10.26. The G_3_ progeny of each G_2R_ parent, present a single point of recombination per sub-QTL Thus, in our example, there will be on average 10.26 points of recombination within each sub-QTL. The corresponding number when polygenic effects are present, is 37.93. These numbers are constants that depend only on N_G3_, and are not affected by C, c, or s. The number of recombination points per sub-QTL when taking polygenic effects into account is quite large, and in view of the simulation results, would seem more than sufficient so as not to be a limiting factor in the precision of setting QTL confidence intervals by the TRP design.

### Simulation

Table 5 shows simulation results with respect to effect of mapping population size (N), number of markers spanning the QTL (k) and marker position within the QTL (L). Results are shown as the Standard error of estimated QTN location relative to the simulated location (SEQTN). Also shown are comparisons of reduction factor (s) as obtained from the simulation (sSIM) and as obtained from the deterministic analysis (sDET). Considering first the effect of marker number, except for N = 1000, at both QTN positions SEQTN is reduced by each step from k = 2 to k = 23, but there is no further decrease in going from k = 23 to k = 50. (For N = 1000 there is a further reduction going from k = 23 to k = 30, but not beyond this). This is somewhat surprising, as we would expect an interaction between population size and marker density. The lack of such interaction is probably due to the fact that number of families was set at 25, so that the number of recombination points across the QTL was a limiting factor in reducing SEQTN by increase in marker density.

Each step increase in N, resulted in a decrease in SEQTN. As could be expected, the marginal decrease in SEQTL was less with each additional step, but still appreciable until the last; the gain in going from N = 9000 to N = 11000 might appear not to be worth the cost. However, this may be a further consequence of the limit of G_2R_ families to 25. In practice, increase in N would be accompanied by an increase in N_G2R_, so that the combined effect of both might be appreciable (see further discussion of Table 6).

**Table 6:** **Standard error of estimated QTN location by simulation, as a function of number of G**_**2R**_ **families (F)** ^1^

**F**	**k = 2**	**5**	**10**	**23**	**30**	**40**	**50**
N = 3000							
500	0.7270	0.2190	0.0889	0.0850	0.0847	0.0841	0.0844
250	0.7420	0.1130	0.0851	0.0863	0.0813	0.0817	0.0819
100	0.4200	0.1120	0.0913	0.0855	0.0851	0.0868	0.0863
50	0.8120	0.2110	0.1250	0.0908	0.0939	0.0957	0.0925
25	0.6800	0.1670	0.1170	0.1030	0.1030	0.1000	0.1020
10	0.8340	0.3410	0.1970	0.1320	0.1270	0.1240	0.1230
N = 11000							
500	0.0669	0.0785	0.0404	0.0298	0.0283	0.0256	0.0237
250	0.0684	0.0784	0.0380	0.0306	0.0285	0.0267	0.0252
100	0.0867	0.0791	0.0412	0.0326	0.0307	0.0293	0.0285
50	0.1030	0.0818	0.0439	0.0347	0.0346	0.0328	0.0306
25	0.1150	0.0843	0.0552	0.0497	0.0490	0.0496	0.0472
10	0.4700	0.1050	0.0896	0.0855	0.0863	0.0855	0.0854

^1^Abbreviations and assumptions. Standard error of estimated QTN location by simulation, as a function of number of G_2R_ families (F) within given total mapping population size, (N), and number of markers spanning the QTL (k). Location of the QTN within its confidence interval (L = 0.57); allele substitution effect at the QTN = 0.2 in standardized units

Placing the QTN closer to the boundary of the QTL (L = 0.77 compared to L = 0.57) gave slightly smaller SEQTL at all population sizes. This was higher for smaller N and smaller k, and decreased as N and k increased. This is an artifact, due to the fact that in the simulation, the estimated QTN position could not fall outside the boundaries of the QTL. Hence, the boundary set an artificial upper limit to the simulated errors, reducing the SEQTN accordingly. However, the effect was small, suggesting that the results for the more central QTN location (L = 0.57) are not affected in a major way by boundary effects.

Table 5 also shows reduction factor (s) achieved at k = 50, F = 25 by the simulation (sSIM) and by the deterministic analysis (sDET). At marker saturation (k = 50), correspondence is rather close for intermediate population sizes (N = 5000 and N = 7000) but fall off to either side. On the low side (N = 1000 and 3000) this is probably due to the aforementioned boundary effect. This affects the simulation results, placing an upper boundary on the error values; but apparently does not affect the deterministic results. On the high side (N = 9000 and 11000) SEQTL for the simulation is probably limited by the small number of G_2R_ parents, and the resulting paucity of recombination points for mapping, while this is not a limitation for the deterministic analyses.

Table 6 shows simulation results with respect to number of G_2R_ families in interaction with population size and number of markers. Generally, there is a clear and major decrease in SEQTN with increase in number of families for given population size. The largest effect on SEQTL is given by the first step, from F = 10 to F = 25, but even at N = 11000, each additional step results in a decrease in SEQTN. SEQTN at F = 500, is just half the SEQTN at F = 25. At N = 3000 there appears to be a slight optimum at F = 250. This may be due to inverse relation of number of families and number of individuals within families. The expected interaction between population size and marker density can be observed: at N = 3000, increase of k from 30 to 50 does not reduce SEQTN; but at N = 11000, from F = 50 on, there is a clear reduction in SEQTN with increased marker density. Thus, as suggested above, by increasing F along with N, major reductions in SEQTN may be achieved, even by going beyond N = 11000. For N = 11000 and F = 50 or more, sSIM is greater than sDET, to an increasing degree with greater F. For example, with F = 500, sSIM = 10.5, while sDET = 7.16. We do not have an intuitive explanation for this effect.

## Discussion

The TRP is aimed at mapping a specific target QTN, previously assigned to a rather broad confidence interval; to a smaller CI, reduced by some desired factor relative to the original CI. As such, it is a Group II design as defined by Darvasi [2]. A TRP mapping population of size 10,000 individuals can reduce CI by a factor of 4 or 5, even for a QTN of relatively small standardized effect (d = 0.2); for a strong QTN (d = 0.5), the same reduction would be provided by a TRP population size under 2000. A two-stage experiment with four-fold reduction at each stage, would reduce a starting CI of 20 cM to 5 cM in the first stage, and to 1.25 cM in the second stage. For d = 0.2, this would require about 16,000 individuals in two cohorts of 8,000 each. For d = 0.5 this would be achieved with two cohorts of about 2000 each. Thus, the TRP design provides a useful solution to the challenge of achieving high resolution mapping for a known specific target QTN. A unique property of the TRP design, is the fact that mapping population size required for a given reduction factor is independent of the size of the starting CI. It will be the same whether the starting CI is 20 cM or 2 cM. Consequently, at a given mapping population size, the smaller the initial CI, the smaller the final CI in proportion. For maximum effectiveness, as shown by the simulation, the TRP design should be implemented with at least 30-40 evenly spaced markers and at least 50 to 100 recombination points within the original CI.

When a QTL is mapped to a large CI, it is not possible to tell if the effect is due to a single QTN in the region, or to the summed effect of a number of closely linked QTN. By dividing the initial CI into sub-intervals, each with considerable mapping power, the TRP design can distinguish between an effect localized to a single point in the original CI, indicating that the total effect was produced by a single QTN, and an effect that is spread all through the QTL, indicating that the total effect represents the summed effects of a number of QTN.

For outcrossing species, WGAS appears to provide a satisfactory solution to the challenge of high resolution mapping of QTN with appreciable effects. This option is obviously not available for high resolution mapping in pure lines of selfing species, or inbred lines of outcrossing species. Although there are a plethora of Group I designs that can provide high resolution mapping across the entire genome (reviewed in Background), with the exception of the AIL design [3] these are all based on specialized resources specifically constructed for high resolution mapping and are perforce limited to the QTL segregating among the founder lines of the resource. For Group II designs that deal with a previously mapped QTL on an *ad hoc* basis, the choice of designs is limited to the ICSC and RPT designs. The TRP design is an addition to this group. Strictly speaking, the NIL design [9] is not a procedure for high or ultra-high resolution mapping, since the isolated donor segments will likely be in the range 10-20 cM. However, any of the NIL sublines, carrying a QTL of interest would be a superb start point for subsequent application of the ICSC, RPT or TRP designs. There is no doubt that when applicable the ICSC design is a very effective means for high resolution mapping, with minimal requirements for population size. The limitation of the ICSC design is the large number of backcross generations required to generate the series of congenic strains spanning the original CI. This is not a major limitation for species such as the mouse with rapid reproduction cycles; but it is a major limitation for the many plant species that have a single growing season a year. Selection within each backcross step for individuals carrying the least amount of donor genome, could probably reduce the number of backcross steps by one or two generations. The RPT design was presented only briefly in [2] and does not appear to have been subjected to detailed analysis, or to have been widely applied. It appears to be very similar to the TRP design proposed and analyzed here. In both designs, individuals that are recombinant within the original CI are chosen as parents of the mapping population. The major difference is that the RPT design works with a limited number of families, each representing one of a laddered series of recombination points spanning the original QTL at 1 cM intervals. Each of the families must be sufficiently powerful to give a decisive decision as to its QTL status and in this way determine whether the QTN is upstream or downstream of the recombination point in that family. In the TRP design, these requirements are relaxed, and the only requirement is that the total number of families is sufficient to give adequate coverage of the original QTL by recombination points, and the total number of recombinant progeny across all families is sufficient for high resolution mapping across the original CI. It should also be noted that the RPT design may have to base on a backcross design in order to obtain the very large numbers required for high power of the individual family, while the TRP design works well with an F_2_ design and fewer individuals per family. Since the F2 design is generally more powerful than the BC design with equivalent numbers (as seen in the present study), this might also favor the TRP. More exact comparison of the two designs remains for deeper analysis of the RPT. However, all in all RPT and TRP can be considered as variants of the same basic design.

## Conclusions

TRP design allows reducing confidence interval of a known target QTL by some desired factor, independent of the original QTL size. The population size required to achieve this depends greatly on the allele substitution effect and also on the polygenic effect and the factor of reduction that was chosen. It is most effective for reducing CI from high resolution (CI = 0.02 - 0.05 Morgan) to ultra-high resolution (0.002 - 0.005 Morgan) since, as opposed to the standard designs, the number of individuals required does not depend strongly on the size of the target CI. Even in the presence of polygenic effects, TRP provides opportunities to achieve CI reduction with a manageable population size where F_2_ and other designs fail. The TRP design also saves greatly in reducing the amount of genotyping required. Thus, the TRP design provides a useful solution to the problem of achieving high and ultra-high-resolution mapping in crosses of inbred or pure lines, where genome wide associations tests are not applicable.

## Appendix

### Calculating N_G3_ for the TRP-BC design

As shown by Weller and Soller [26] if M_1_ is a marker located at an estimated QTN location, the probability that the CI of QTN location includes the marker M_2_ located at a remove of L Morgan from M_1_, is equal to the probability of obtaining the value

$$ {\mathrm{Z}}_{\upalpha /2}=\mathrm{D}/\mathrm{S}\mathrm{E}\left(\mathrm{D}\right), $$

where ,

Z_α/2_ is the standard normal variable corresponding to a probability of α/2,

D = E(M_1_) - E(M_2_), where E(M_1_) is the expected effect at M_1_ (QTN located at the marker), E(M_2_) is the expected QTN effect at M_2_ (QTL located at a remove from the QTN) considering recombinant individuals only; and SE(D) is the standard error of D.

From Table 7 it is apparent that there are two marker genotype groups, each of which is composed of a single recombinant marker group. Marker genotype-group M_1_m_1_ is composed of recombinant genotype-group A of Table 7 with genotype: M_1_m_2_/m_1_m_2_, having genotypic value h; frequency 1/4 of the entire G3 population. Marker genotype-group M_2_m_2_ is composed of recombinant genotype-group B of Table 7 with genotype: m_1_M_2_/m_1_m_2_, having genotypic value -d; frequency 1/4 of the entire G3 population.

**Table 7:** **Composition of G**_**3**_ **population for TRP-BC design** ^**1**^

**G**_**3**_ **progeny**	**G2R parent**	
	M_1_m_2_/m_1_m_2_	m_1_M_2_/m_1_m_2_
Class I	M_1_m_2_/m_1_m_2_	m_1_M_2_/m_1_m_2_
	1/4 h A	1/4 -d B
Class II	m_1_m_2_/m_1_m_2_	m_1_m_2_/m_1_m_2_
	1/4 -d NR	1/4 -d NR

^1^Each cell shows a G_3_ progeny group according to Class and the G_2R_ parent, showing: Marker genotype of the progeny group (above); proportion of the progeny group in the total G_3R_ population (below-left); genotypic value of the progeny group (below-center); Code designation (A, B) of the progeny group (below-right). Class I, Heterozygous recombinant progeny; Class II, Homozygous non-recombinant progeny; NR, non-recombinant progeny group not included in the G_3R_ mapping population; d, allele substitution effect in standardized units; h, degree of dominance.

Letting italics denote the mean genotypic value of the corresponding marker genotype group (including recombinant genotypes only), we have

$$ \mathrm{E}\left(\mathrm{M}1\right) = {M}_1{m}_1\mathit{\hbox{-}}{m}_1{m}_1 $$

$$ \mathrm{E}\left(\mathrm{M}2\right) = {m}_2{M}_2\mathit{\hbox{--}}\ {m}_2{m}_2 $$

A1 $$ \mathrm{D} = \left({M}_1{m}_1\mathit{\hbox{-}}\ {m}_1{m}_1\right) - \left({m}_2{M}_2\mathit{\hbox{--}}\ {m}_2{m}_2\right) $$

On this basis, letting *A, B,* represent the genotypic values of the corresponding genotype groups, we have

$$ {M}_1{m}_1 = A\ \mathit{\hbox{-}}\ B $$

$$ {M}_2{m}_2=B\mathit{\hbox{-}}\ A $$

Substituting in (1) and combining like terms, we obtain

$$ \mathrm{D} = \left(A - B\right)\ \hbox{--}\ \left(B\ \hbox{--}\ A\right) = 2\left(A\ \hbox{--}\ B\right) $$

Substituting genotypic values of the recombinant groups we have

$$ \mathrm{D} = 2\left(\mathrm{h}\ \hbox{--}\ \left(\hbox{-} \mathrm{d}\right)\right) = 2\ \left(\mathrm{h}+\mathrm{d}\right) $$

To calculate SE(D), we assume that variance within the G_3_ families is the same as variance within the F_2_ generation (set to 1.0 for standardization). This is conservative, as the genetic variance within G_3_ BC families will be less than in an F_2_ population, depending on degree of homzoygosity in their G_2R_ parent. On this basis, we can write:

$$ {\sigma^2}_{\mathrm{A}}={\sigma^2}_{\mathsf{B}} = 1/\left({\mathsf{N}}_{\mathsf{G3}}/\mathsf{4}\right)=\mathsf{4}/{\mathsf{N}}_{\mathsf{G3}} $$

(as each of these genotype groups comprises 1/4 of the G_3_ mapping population) of size N_G3_

Then,

$$ \mathrm{S}{\mathrm{E}}^2\left(\mathrm{D}\right) = 4\left(4/{\mathrm{N}}_{\mathrm{G}3} + 4/{\mathrm{N}}_{\mathrm{G}3}\right) = 32/{\mathrm{N}}_{\mathrm{G}3} $$

Substituting, we have

$$ {Z}_{a/2} = 2\left(\mathrm{h}+\mathrm{d}\right).\ \mathrm{Assuming}\ \mathrm{h} = 0,\ \mathrm{we}\ \mathrm{h}\mathrm{ave} $$

$$ {Z}_{a/2} = 2\mathrm{d}/{\left(32/{\mathrm{N}}_{\mathrm{G}3}\right)}^{0.5}\kern.4em \mathrm{and}\ \mathrm{solving}\ \mathrm{f}\mathrm{o}\mathrm{r}\ {\mathrm{N}}_{\mathrm{G}3}\mathrm{we}\ \mathrm{o}\mathrm{btain} $$

$$ {\mathrm{N}}_{\mathrm{G}3} = 8{{\mathrm{Z}}_{\mathrm{a}/2}}^2/{\mathrm{d}}^2 $$

If we set α=0.05 and d=0.2, as we did for the TRP-F2 design, we have Z_α/2_=1.96, and

$$ {\mathrm{N}}_{\mathrm{G}3} = 768.3 $$

Since the interval between M_1_ and M_2_ defines only half of the CI of QTN location, to cover the entire CI would require twice this

$$ {\mathrm{N}}_{\mathrm{G}3}=16{{\mathrm{Z}}_{\upalpha /2}}^2/{\mathrm{d}}^2 = 1536.6 $$

On these assumed values for α and d, the basic G_3R_ population required for CI(0.95) = N_G3_= 1536.64 This number is constant for given d, and α, and does not depend on the target size of the confidence interval. The corresponding value for TRP-F_2_ design is 512.34. On general principles we would have expected NG3 for TRP-BC with h=0 to be twice that for TRP-F_2_ (that is, 1024,68). The difference is due to the fact that the mapping population for the TRP-F_2_ is 75% of the total population; while the mapping population for the TRP-BC is 50% of the total population. If we increase the TRP-BC N_G3_ value by 50% to make up for this we obtain NG3= 1529.37, as found.

## Abbreviations

QTN: Quantitative Trait Nucleotide
QTL: Quantitative Trait Locus
TRP: Targeted recombinant Progeny
CI: Confidence Interval
MAS: Marker assisted selection
MAI: Marker assisted introgression
SNP: Single Nucleotide Polymorphism
WGAS: Whole genome association studies
LD: Linkage disequilibrium
AIL: Advanced intercross line
HS: Heterogeneous Stock
RIL: Recombinant Inbred Lines
MAGIC: Multi-parent Advanced Generation Inter-Cross
RPT: Recombinant Progeny Testing
ISCS: Interval-Specific Congenic Strains
g.d.p.: genotyping data points
SEQTN: Standard error of QTN
